# Combined *Rhodiola rosea* and eccentric training boost endurance performance and lower-limb reactive strength in recreationally active women

**DOI:** 10.3389/fphys.2025.1663086

**Published:** 2025-09-08

**Authors:** I-Lin Wang, Yu Su, Long Zhang, Maobing Tang, Yi-Ming Chen

**Affiliations:** ^1^ School of Physical Education, Liupanshui Normal University, Liupanshui, Guizhou, China; ^2^ Hebei Rehabilitation Center, Hebei General Hospital, Shijiazhuang, Hebei, China; ^3^ Department of Basic Education, Tarim Institute of Technology, Alaer, Xinjiang, China; ^4^ Department of Food Science, Fu Jen Catholic University, New Taipei City, Taiwan; ^5^ Ph. D. Program in Nutrition and Food Science, Fu Jen Catholic University, New Taipei City, Taiwan

**Keywords:** eccentric fly-wheel training (ECC), salidroside supplementation, reactive strength index (RSI), muscle-damage biomarkers, endurance performance

## Abstract

This study aimed to determine whether chronic *Rhodiola rosea* (salidroside) supplementation augments neuromuscular and metabolic adaptations to flywheel eccentric (ECC) training. In total, 30 recreationally active female students were randomly assigned to (1) sedentary placebo control (SC, n = 10), (2) ECC training + placebo (ECC, n = 10), or (3) ECC training + salidroside (150 mg day^−1^; ECC + SA, n = 10) for 4 weeks. Pre- and post-intervention assessments included exhaustive cycling time to volitional fatigue, peak oxygen uptake (VO_2_ max), drop vertical jump (DJ) reactive strength index (RSI), and blood biomarkers of muscle damage and metabolism. ECC and ECC + SA prolonged time to exhaustion by 33% and 45%, respectively, without altering VO_2_ max. Across DJ_1_–DJ_160_, RSI significantly increased in both ECC groups (*p* < 0.01). Salidroside conferred an additional 8%–33% RSI advantage during the final 40 contacts (DJ_120_–DJ_160_; *p* < 0.05). The ECC + SA group exhibited lower postcycling creatine kinase (CK) and higher free fatty acids (FFA) than the SC group (*p* < 0.05). Triacylglycerol and low-density lipoprotein cholesterol decreased in ECC + SA compared with SC (*p* < 0.05), whereas body composition remained unchanged. In conclusion, four weeks of ECC training improved endurance and stretch–shortening performance. Salidroside further enhanced late-stage RSI, with additive antifatigue and muscle-protective effects during high-load eccentric conditioning in recreationally active women.

## 1 Introduction

The genus *Rhodiola* has been found to contain salidroside (SA), which could alleviate effectively prevent the lack of oxygen supply to the brain after high-altitude training (Liao et al., 2019). Interestingly, previous animal studies have found that *Rhodiola rosea* supplementation could improve aerobic exercise ability and that a single *Rhodiola* supplement can reduce lactic acid produced during exercise, thereby reducing fatigue and improving aerobic exercise ability (Wei et al., 2017). Moreover, another study found that short-term *R. rosea* supplementation for 3 days increased the average anaerobic capacity, average anaerobic capacity, and average peak power compared to placebo following repeated Wingate anaerobic tests (Ballmann et al., 2019). *R. rosea* has been found to exert antioxidant activity, with researchers finding that long-term intake of *R. rosea* can potentially aiding recovery in oxidative stress heavy sports among healthy men (Jówko et al., 2018). Furthermore, studies have shown that the polyphenol content in *Rhodiola* extract exerted antioxidant activity against ROS produced during muscle contraction, suggesting that *R. rosea* root extract reduced oxidative stress caused by muscle contraction and restored ROS production to physiological levels (Xu and Li, 2012).

Notably, several studies have reported negligible or inconsistent ergogenic effects of R. rosea on resistance or sprint performance, underscoring the need to delineate context-specific efficacy, such as exercise mode, training status, dose and duration. Our study addresses this gap by examining SA during eccentric conditioning and prolonged stretch–shortening cycle tasks (Sanz-Barrio et al., 2023; Tinsley et al., 2024). Resistance training has been shown to increase muscle strength and hypertrophy. Traditional resistance training protocols use consecutive eccentric (ECC) and concentric (CON) cycles. However, previous studies have highlighted the importance of the eccentric phase for increasing muscle time under tension, which enhances muscle strength (Coratella and Schena, 2016). In this study, eccentric training (ECC) was performed using flywheel equipment (K-box4, Exxentric ABTM, Bromma, Sweden), which has been shown to isolate and increased eccentric overload (Tesch et al., 2004). Skeletal muscles produce more force when performing eccentric contractions (Jiménez-Jiménez et al., 2008). However, traditional resistance eccentric training had failed to take advantage of muscle eccentric contractions given the difficulty of increasing the weight during the eccentric phase with traditional methods (Hortobágyi, 2003; Norrbrand et al., 2010). Nonetheless, when accelerating a flywheel at the concentrate or lifting phase and decelerating it in a short period of time, a higher eccentric force will have to be generated, making flywheel ECC training the most effective method for increasing muscle power (Fernandez-Gonzalo et al., 2014; Maroto-Izquierdo et al., 2017). Because eccentric contractions generate greater force at a lower metabolic cost and flywheel systems allow controlled eccentric overload, we adopted an eccentric-emphasized protocol to amplify muscle damage and antifatigue phenomena and to reduce variability inherent in mixed concentric eccentric schemes.

Eccentric muscle contractions cause substantially greater skeletal muscle damage than do concentric or isometric contractions (Gottschalk et al., 2025). High-intensity eccentric work, wherein the external loads exceed the force produced by the muscle, disrupts sarcomere integrity, ruptures myofibers and connective tissue, reduces contractile capacity, elicits delayed-onset muscle soreness, and accelerates protein catabolism. These events are accompanied by a marked increase in serum creatine kinase (CK), proinflammatory cytokines, and ROS, ultimately impairing subsequent exercise performance (James et al., 2024; Gottschalk et al., 2025). Intermittent downhill (eccentric-biased) running further activates Nuclear Factor Kappa-light-chain-enhancer of Activated B cells (NF-κB) signaling, which regulates the expression of genes required for tissue regeneration (Minari et al., 2022).

Despite the documented clinical and ergogenic effects of *R. rosea*, little is known about how its principal bioactive constituent, SA, interacts with eccentric training to influence muscle damage and functional performance. Therefore, the present study investigated whether supplementation with *R. rosea* extract for 4 weeks modulates biochemical markers of muscle injury and improves physical performance during and after a 4-week program involving repetitive eccentric exercise among recreationally active females. We hypothesised that (i) eccentric training would increase time-to-exhaustion and reactive-strength index (RSI) relative to a sedentary control, and (ii) salidroside co-supplementation would further (a) attenuate the post-exercise rise in creatine kinase and blood ammonia and (b) augment late-stage RSI during repeated drop jumps. Flywheel eccentric loading causes significant sarcomere disruption, ROS production, and neuromuscular fatigue. Salidroside’s antioxidant and anti-fatigue properties provide a targeted countermeasure for the mechanical and metabolic stresses of high-load eccentric exercise, an area unexplored in previous (Choi, 2014).

To minimize sex related variability in neuromuscular and metabolic adaptations to eccentric loading and salidroside supplementation, only recreationally active women were recruited; however, this limits the generalizability of findings to male populations.

## 2 Materials and methods

### 2.1 Peparation of R. rosea extract

*R. rosea* root extract, standardized to *SA*, was prepared according to a previously published protocol (Liu et al., 2021). Both extraction and drying were performed at Professor Yi-Ming Chen’s laboratory. The placebo comprised isocaloric corn-starch powder packed in identical capsules, rendering the two interventions visually indistinguishable to both participants and investigators.

### 2.2 Participants

An *a priori* power analysis was performed in G*Power v3.1.9.7. Assuming a medium effect size (Cohen’s d = 0.50) for the primary outcome (time-to-exhaustion), α = 0.05 (two-tailed) and power = 0.80, a minimum of 24 participants per group was indicated. However, because the participant pool was restricted to students enrolled in a single physical-education course, we were able to recruit only 30 eligible recreationally active women*, resulting in smaller group sizes than the a priori target.* Participants were classified as recreationally active based on *a priori* criteria. All volunteers completed the Physical Activity Questionnaire-Short Form (IPAQ-SF, and eligibility required ≥3 sessions·week^−1^ of structured moderate-to-vigorous activity (≥30 min per session; Borg RPE ≥12) over the preceding 3 months, with no involvement in varsity/elite or professionally supervised training. The sample reported [1605 ± 332] MET·min·week^−1^. Common activities included jogging, group aerobic classes, recreational ball games, and cycling for transport. None had prior experience with flywheel-based or formal plyometric programs. This level of activity aligns with a Division III/club sport profile habitually active but not undergoing high-volume, periodized conditioning.

Utilizing a randomized, double-blind, parallel-group design, this study included a total of 30 healthy female college students enrolled in a physical education course and randomly allocated them to one of three groups: a sedentary control group that received supplementation with dextrose placebo powder (SC), the eccentric exercise group that received supplementation with placebo (ECC), and the eccentric exercise group that received supplementation with SA (ECC + SA). All three groups showed no significant difference in age, height and weight (age = 19.0 ± 1.4 years; height = 165.0 ± 3.9, 164.6 ± 6.8, and 164.7 ± 4.8 cm; weight = 56.8 ± 6.0, 56.9 ± 10.3, and 57.0 ± 10.3 kg, respectively). None of the participants had any record of cardiovascular or respiratory disease or musculoskeletal injury. Participants who had trained in any jumping program were excluded. All study procedures were approved by the Joint Institutional Review Board of Chinese Clinical Trial Register (ChiCTR1900027683). This study was conducted in accordance with the ethical principles stated in the Declaration of Helsinki. After enrollment, both SA and placebo powders were encapsulated in soft pine bark capsules. Each group (n = 10) consumed 150 mg/day of SA or placebo supplements before breakfast for 4 weeks. The ECC and ECC + SA groups came to our laboratory every day for eccentric exercise assessment, with both groups performing the same number of sets at the same intensity (i.e., percentage body weight). Besides ECC training, all subjects were asked to maintain normal activity and refrain from any strenuous exercise. Subjects participate 2 study visits on Day 0 (pretest) and Day 28 (posttest). After the 4-week supplementation, a posttest was conducted. At posttest, participants guessed capsule identity (SC vs. SA); correct identification did not differ from chance (χ^2^ = 0.27, *p* = 0.60), confirming successful blinding.

Participants were instructed to maintain habitual intake at approximately 2,200–2,300 kcal·day^-1^, based on an estimated activity level of 1.6–1.7 (1650 MET·min·wk^−1^). The target macronutrient distribution was 55% carbohydrates, 20% protein, and 25% fat.”

### 2.3 Anthropometric measurements

At the beginning and end of the experiments, the body weight (kg), body height (cm), body mass index (BMI; kg/m^2^) fat-free mass (FFM, kg), body fat percentage (% of body weight), and water content percentage (%) of all 30 participants were measured using a body fat analyzer (MC-980, Tanita Co., Tokyo, Japan) based on a previously reported method (Chen et al., 2021).

### 2.4 Eccentric exercise protocol

Eccentric exercise training was performed using the flywheel equipment (K-box4, Exxentric ABTM, Bromma, Sweden). Before the training period, all participants completed the training sessions to familiarize themselves with the training process, including a review of the safety criteria and practice with squat training using a flywheel gravity device with an inertia of 0.01 kg/m^2^. The tension band of the K-box4 device was adjusted according to the height of individual participants, with the squat range of motion being standardized for all participants. The length of the tension band was used to determine the range of motion of the knee joint angle for all subjects. Before starting the training program, all participants performed a 10-min warm-up. During the training program, participants performed 15 repetition maximum of deep flywheel squats with a speed of 0.01 kg/m^2^ inertia load. The range of motion of the participants was standardized, and a tape was used to ensure that the knee angle reached 90 degrees (thighs parallel to the ground). Consequently, the participants performed squats by extending their knees (180 degrees) from a low (90 degrees) position. Ankle extension was not allowed. Participants were instructed to perform the concentric action phase as quickly as possible while delaying braking until the last part of the eccentric phase. Throughout the training program, the participants were given loud verbal encouragement. The training was designed to overload the eccentric phase using the flywheel (K-Box4). Although a brief concentric action is required to accelerate the wheel, the primary stimulus was the deceleration phase, during which participants were instructed to delay braking to maximize eccentric loading.

### 2.5 Exhaustive endurance exercise and biochemical tests

The exhaustive cycling test was performed at the 26th day of the intervention. To avoid the effects of dietary intake on outcome measures during testing days, subjects ate a standardized breakfast on the morning of the test day. The standard breakfast comprised 55% carbohydrates, 15% protein, and 30% lipids, for a total caloric intake of 380 kcal. Tests were started 30 min after all participants consumed the supplement 30 min. Exhaustive tests were performed using a face mask on a cardiopulmonary ergospirometry device. Subjects were instructed to avert any strenuous exercise activity for 3 days before the biking exercise test for VO_2_max analysis. The speed and wattage of the bike were based on the ramp protocol on a cycle (Michalik et al., 2019). VO_2_ and VCO_2_ were measured breath by breath via calibrated metabolic cart (Schiller CS200, Schiller AG, Switzerland). The ramp protocol increased workload by 15 W min^−1^ until volitional exhaustion. VO_2_ max was defined as the highest 30s averaged VO_2_ accompanied by at least two of the following: RER ≥1.10, HR ≥ 90% of age-predicted maximum, or a VO_2_ plateau (<150 mL min^−1^ increase despite workload increment). After the exhaustive endurance exercise challenge, lactate, ammonia, glucose, CK, lactate dehydrogenase (LDH), and free fatty acid (FFA) levels were assessed using a Beckman Coulter AU5800 autoanalyzer (Beckman Coulter Inc., Brea, CA, United States). At the end of the experiment (30 days), all subjects had fasted for at least 8 h and took the last supplementation dose, after which serum biochemical indices were assessed using a Beckman Coulter AU5800 autoanalyzer (Beckman Coulter Inc., Brea, CA, United States).

### 2.6 Endurance drop jumps (DJs) with kinetic and kinematic analysis

The total number of 160 jumps was selected based on protocols used in prior fatigue studies aiming to observe longitudinal decrements in explosive performance and stretch-shortening cycle function. Although this setup does not mimic a single real-game scenario, it maintains ecological validity by reproducing the intensity and density of jump tasks common in sport-specific training and match play. Additionally, previous studies investigating the effects of nutritional or ergogenic supplementation on neuromuscular performance have also adopted similar high volume DJ protocols (160 jumps) to reliably induce fatigue and assess intervention efficacy (Chen et al., 2021). All participants wore standardized shoes and shorts and completed a familiarization session before data collection. After a dynamic warm-up (5 min of treadmill running at 6 km h^-1^ plus lower-limb stretches), the participants rested for 5 min and then performed three countermovement jumps (CMJs) on dual force plates. During each CMJ, the participant began from an upright stance, descended to ∼90° knee flexion, and finished with a maximal vertical takeoff while maintaining arm swing, with each attempt separated by 60 s of passive recovery. The highest CMJ was defined as 100% jump height (H). Target drop-jump heights were set at 70%, 100%, and 130% of H. Notably, 130% H was selected as the test height after 3 min of rest and five familiarization trials. Subjects then completed 160 drop vertical jumps at this height with 10 s between jumps, subsequently landing and rebounding on the force plates with the hands placed on the waist to eliminate arm assistance. Data processing was performed using a custom MATLAB program (version 2019a, MathWorks Inc., Natick, MA, United States). A fourth-order low-pass Butterworth digital filter with a cutoff frequency of 50 Hz can smooth the ground reaction forces and marker trajectories. The intervention effects were assess using the parameters of the reactive strength index, which was calculated using the following formula (Ward et al., 2019; Wang et al., 2021):

$$\text{Reactive strength index }\left[\text{RSI}=\frac{\text{JH}}{\text{Contact}\;\text{time}}\right],$$

where contact time was defined as the duration from initial contact, at which the vertical ground reaction force exceeded a threshold of 10 N, to toe-off in an instant. RSI can represent the response speed of the participants from touchdown to takeoff.

### 2.7 Statistical analysis

Statistical analyses were performed using MATLAB software (version 2019a, MathWorks Inc., Natick, MA, United States). All data were expressed as means ± SD. One-way analysis of variance (ANOVA) followed by *post hoc* Tukey’s HSD tests was performed to determine significant differences in biochemistry and exhaustive exercise tests results between multiple groups. A mixed-design two-way ANOVA, with supplementation and time as independent variables, was used to compare pretest and posttest variables. A *post hoc* independent repeated *t*-test was used to determine the significance of the main and interaction effects. For all tests, a *p* value of <0.05 was considered significant. To assess the magnitude of differences, effect sizes were calculated using Cohen’s d. For between-group comparisons, Cohen’s d was computed based on pooled standard deviations. The interpretation of d was as follows: small (0.2 ≤ d < 0.5), medium (0.5 ≤ d < 0.8), and large (d ≥ 0.8). Effect sizes were reported alongside *p*-values to provide additional insight into the practical significance of the findings.

## 3 Results

### 3.1 Effects of ECC training or ECC + SA on anthropometric measurements

Anthropometric measurements are summarized in Table 1. A total of 30 participants underwent body composition measurements on before (pretest, day 0) and after (posttest, day 28) the intervention. Participants in the placebo, ECC and ECC + SA groups had a height of 165.0 ± 3.9, 164.6 ± 6.8, and 164.7 ± 4.8 cm, respectively. No significant differences in height were observed between each group. Aside from height, pretest measurements of body weight, fat mass (FM), FFM, BMI (kg/m^2^), and water content did not differ between the groups. For posttest measurements, no significantly difference in body weight, FM, FFM, BMI, and water content were observed between the SC, ECC, and ECC + SA groups. Thus, these findings suggest that the 4-week ECC and ECC + SA interventions did not influence body composition. Focusing on motion analysis, biochemistry tests, and exercise performance test could yield more precise results.

**TABLE 1:** General characteristics of body composition in sedentary control (SC), eccentric training (ECC), and eccentric training combined with salidroside supplementation (ECC + SA) groups (n = 10 subjects/group).

Characteristic	Treatment	Pretest	Posttest	*p* values	
				Pretest	Posttest
Weight (kg)	SC	56.8 ± 6.0	57.9 ± 4.8	0.9985	0.6243
	ECC	56.9 ± 10.3	55.3 ± 9.5		
	ECC + SA	57.0 ± 10.3	55.5 ± 4.6		
FM %	SC	29.1 ± 7.5	30.8 ± 6.3	0.9848	0.4141
	ECC	29.1 ± 5.8	28.0 ± 4.3		
	ECC + SA	28.9 ± 3.9	28.4 ± 4.0		
FFM (kg)	SC	41.14 ± 1.8	40.5 ± 2.3	0.8831	0.8144
	ECC	40.38 ± 4.5	40.6 ± 4.6		
	ECC + SA	40.44 ± 4.4	41.5 ± 4.7		
BMI (kg/m^2^)	SC	20.97 ± 3.3	22.0 ± 3.3	0.9227	0.2322
	ECC	20.65 ± 2.3	20.1 ± 2.2		
	ECC + SA	20.54 ± 1.5	20.9 ± 1.5		
Water content (%)	SC	28.54 ± 2.0	27.6 ± 1.0	0.9141	0.9207
	ECC	27.95 ± 3.4	28.0 ± 3.8		
	ECC + SA	28.43 ± 4.2	27.5 ± 2.6		

Data are presented as mean ± standard deviation. The posttest was conducted 4 weeks after ECC training or ECC training combined with SA supplementation. Statistical analysis was performed using one-way ANOVA. Data in the same row (within group) followed by different letters (a, b) were significantly different at *p* < 0.05.

### 3.2 Effects of ECC training or ECC + SA supplementation on physiological characteristics after the exhaustive cycling test

**FIGURE 1:**
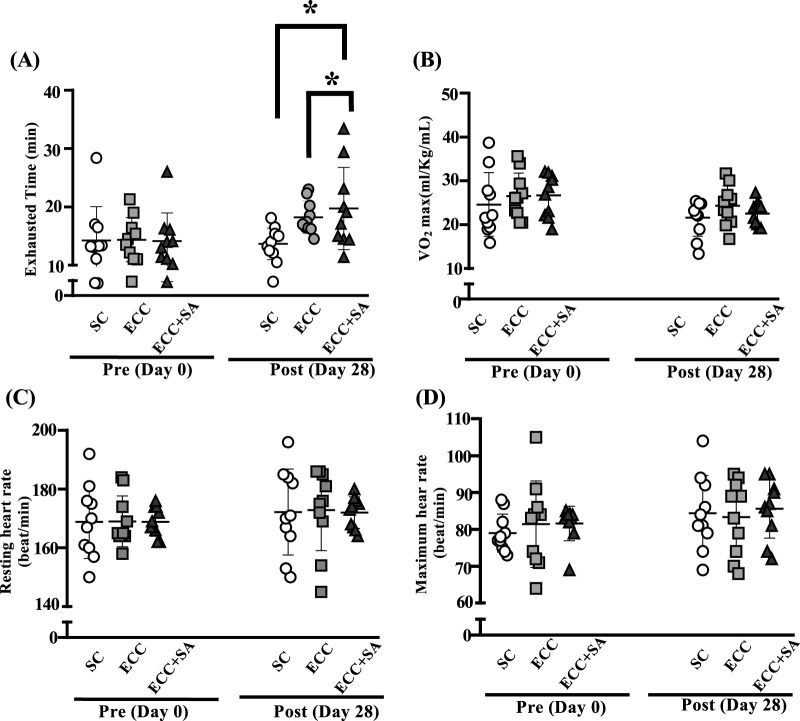
Effects of a 4-week eccentric (ECC) training and eccentric training combined with salidroside supplementation (ECC + SA) on **(A)** time to exhaustion, **(B)** maximal oxygen consumption (VO_2_max), **(C)** resting heart rate, **(D)** maximum heart rate after an exhaustive biking challenge. Subjects performed either ECC training and ECC + SA for 4 weeks and 1 h following the last administration, after which an exhaustive exercise challenge test was conducted. Data are expressed as mean ± standard deviation with 10 subjects in each group. An asterisk (*) indicates a statistically significant difference in posttest measurements (*p* < 0.05).

Before ECC training or ECC + SA supplementation, endurance exercise test performance was measured through an exhaustive cycling pretest (Figure 1). After 4 weeks of ECC training or ECC + SA supplementation, endurance exercise test performance was measured using an exhaustive cycling test. As shown in Figure 1A, the SC, ECC, and ECC + SA groups had a time to exhaustion of 14.3 ± 5.8, 14.4 ± 3.8, and 14.1 ± 4.8 min, respectively, in the pretest. No significant differences in time to exhaustion was observed between each group at pretest. At the posttest, the SC, ECC, and ECC + SA group showed a time to exhaustion of 13.7 ± 2.7, 18.2 ± 2.8, and 19.7 ± 7.0 min, respectively. Notably, time to exhaustion was 24.95% (ES = 1.64, *p* = 0.0375) and 30.80% (ES = 1.13, *p* = 0.0069) higher in the ECC and ECC + SA groups than in the SC group in the posttest. Figure 1B shows the pretest and posttest results of the maximal oxygen consumption (VO_2_max) test in the SC, ECC, and ECC + SA groups. No significant differences in both pretest and posttest results were observed between each group (*p* = 0.6857, pretest and *p* = 0.3009, posttest). However, both the initial and maximum heart rates during the exhaustive cycling exercise test did not significantly differ between each group in the pretest and posttest (*p* = 0.7147 pretest and *p* = 0.8630 posttest, respectively; *p* = 0.9996 pretest and *p* = 0.9847 posttest, respectively; Figures 1C,D). Thus, our results demonstrated that 4 weeks of ECC training and ECC + SA supplementation prolonged the exhaustion time but did not enhance the oxygen consumption compared to the SC group. Overall, SA supplementation could improve endurance exercise performance during the eccentric training period.

### 3.3 Effects of ECC training or ECC + SA supplementation on repeated DJ performance

**FIGURE 2:**
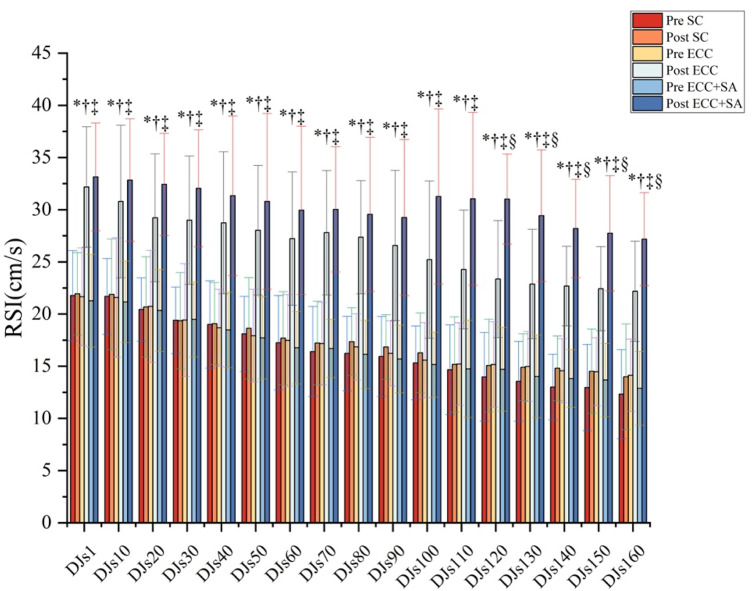
Reactive strength index (RSI) parameters during repeated drop-jump (DJ) testing after 4 weeks of eccentric training (ECC) with placebo or with salidroside supplementation (ECC + SA). “*” indicates a significant group × time interaction; “†” Indicates a significant difference between the pretest and posttest results in the ECC group; “‡” indicates a significant difference between the pretest and posttest results in the ECC + SA group; “∫” indicates a significant difference between the ECC and SC groups at the posttest; “£” indicates a significant difference between the ECC + SA group and SC group at the posttest; “§” indicates a significant difference between the ECC and ECC + SA groups at the posttest (*p* < 0.05). Data are presented by mean ± standard deviation.

As shown in Figure 2, two-way ANOVA revealed significant group × time interaction (all *p* ≤ 0.006) for the RSI from DJ1 to DJs160. Compared to the pretest results, pairwise comparison showed that the RSI in the ECC and ECC + SA groups increased significantly by 41% to 64% (ES = 1.01–1.59) and 55% to 111% (ES = 1.28–3.87), respectively, at the posttest (all *p* ≤ 0.011) after DJ1–DJs160. However, no significant difference between the pre- and posttest RSI was observed in the SC group. At the posttest, the RSI in the ECC and ECC + SA groups increased significantly by 41% to 61% (ES = 1.39–2.27, all *p* ≤ 0.044) and 50% to 106% (ES = 1.77–3.64, all *p* ≤ 0.012), respectively, after DJ1–DJs160 compared to the SC group. Specifically, compared to the ECC group, the ECC + SA group showed a significant increase in RSI (33%, 29%, 24%, 24%, and 23%, respectively) after DJs120 (ES = 1.53, *p* = 0.014), DJs130 (ES = 1.13, *p* = 0.030), DJs140 (ES = 1.29, *p* = 0.045), DJs150 (ES = 1.10, *p* = 0.021), DJs160 (ES = 1.08, *p* = 0.031) at the posttest. Thus, our findings suggested that an eccentric training protocol can increase the reactive strength index, but SA supplementation yielded an additional statistically significant gain that was most pronounced at the highest cumulative plyometric loads, implying that SA enhances explosive stretch–shortening cycle performance.

### 3.4 Effects of ECC training or ECC + SA on lactate, ammonia, CK, glucose, LDH, and FFA after the exhaustive exercise test

**FIGURE 3:**
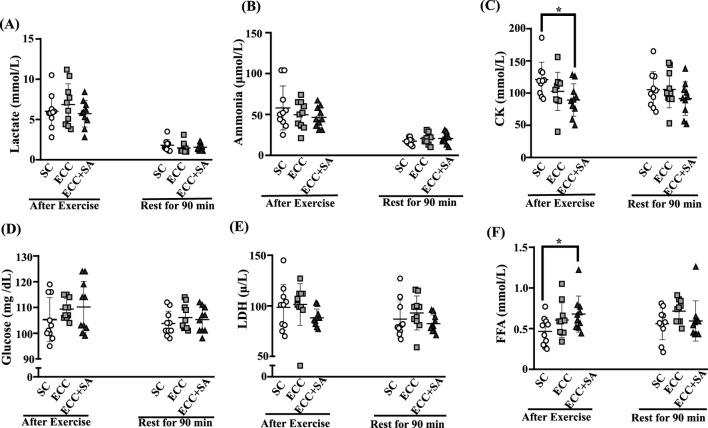
Effects of the 4-week ECC or ECC + SA supplementation on **(A)** lactate, **(B)** ammonia, **(C)** creatine kinase (CK), **(D)** glucose, **(E)** lactate dehydrogenase (LDH), and **(F)** free fatty acid (FFA) levels were 0.47 ± 0.17, 0.61 ± 0.21, and 0.68 ± after the exhausted cycling test and resting for 90 min. All participants consumed a standard breakfast comprising 55% carbohydrates, 15% protein, and 30% lipids, for a total calorie intake of 380 kcal. Asterisk (*) indicates a statistically significant difference between the measurements after exercise and after resting for 90 min.

Biochemical analysis was used to determine the capacity for fatigue recovery in the SC, ECC, and ECC + SA group after the cycling test (Figure 3). Tests were conducted at three points: before and after acute exhausted cycling test (data before the cycling test not shown, no significant difference was found between each group, *p* > 0.05) and then after 90 min of rest after the cycling. In Figure 3A, lactate levels after exercise were 6.02 ± 2.08, 6.84 ± 2.60 and 5.72 ± 1.64 mmol/L in the SC, ECC, and ECC + SA groups, respectively. After 90 min of rest, lactate levels were 1.80 ± 0.72, 1.47 ± 0.66, and 1.56 ± 0.45 mmol/L in SC, ECC, and ECC + SA groups, respectively. No significant differences in lactate levels were observed between each group both after acute exercise and rest for 90 min (*p* = 0.4896 and 0.4776, respectively). Ammonia levels after exercise (Figure 3B) were 57.9 ± 26.9, 49.7 ± 16.7, and 46.2 ± 12.6 μmol/L in the SC, ECC, and ECC + SA groups, respectively. After 90 min of rest, ammonia levels were 17.4 ± 3.8, 20.5 ± 6.7, and 19.7 ± 9.7 (μmol/L) in the SC, ECC, and ECC + SA groups, respectively. No significant difference in ammonia levels were noted at both time points (*p* = 0.4162 and 0.6109, respectively). CK levels (Figure 3C) were 121 ± 27, 103 ± 30, and 89 ± 25 U/L in the SC, ECC, and ECC + SA groups, respectively, with significant differences having been observed after exercise (*p* = 0.0496). CK levels in the ECC + SA group decreased significantly by 26.20% (ES = 1.23, *p* = 0.0155) compared to that in SC group after exercise. After 90 min of rest, however, no significant differences in CK levels were observed between each group (*p* = 0.4362). As shown in Figure 3D, no significant differences in glucose levels were observed between each group after exercise (*p* = 0.3465) and 90 min of rest (*p* = 0.5716). Figure 3E revealed no significant differences in LDH levels at both time points (*p* = 0.2573 and 0.3194, respectively). FFA levels were 0.47 ± 0.17, 0.61 ± 0.21, and 0.68 ± 0.22 in the SC, ECC, and ECC + SA groups, respectively, after the cycling test. The ECC + SA group showed a significant 31.37 %increase in FFA levels (ES = 1.10, *p* = 0.0271) compared to the SC group after the cycling test. After 90 min of rest following the cycling test, no significant differences in FFA levels were observed between each group (*p* = 0.2032). These finding indicate that 4 weeks of ECC training could increase the time to exhaustion during cycling and may improve exercise economy but promoting increased fat utilization as a fuel source (FFA increase). Moreover, SA + ECC could reduce the muscle fatigue index (CK level) and enhance energy transport (FFA level) after endurance exercise.

### 3.5 Biochemical indices after the 4-week ECC training or ECC + SA supplementation

After the 4-week ECC or ECC + SA intervention, health-related biochemical data were analyzed (Table 2). Notably, no significant differences in the pretest data were observed between each group (data not shown). Serum levels of AST, ALT, CK ALB, BUN, creatinine, glucose, total cholesterol (TC), and high-density lipoprotein (HDL) did not significantly differ between each group. Concerning the lipid profile, except for TC and HDL levels, TG levels decreased significantly by 36.82% (ES = 0.87, *p* = 0.0261) in the ECC + SA group compared to that in the SC group. Low-density lipoprotein (LDL) levels decreased significantly by 16.81% (ES = 0.9, *p* = 0.0295) and 15.10% (ES = 0.80, *p* = 0.0481) in both the ECC and ECC + SA groups compared to the SC group. Thus, our findings showed that both ECC training and SA supplementation had beneficial effects on the lipid profile.

**TABLE 2:** Biochemical analysis of the ECC training and ECC training + SA supplementation groups at the end of the experiment.

Parameter	SC	ECC	ECC + SA
AST (U/L)	16.20 ± 4.78	16.20 ± 2.82	13.80 ± 1.93
ALT (U/L)	16.10 ± 12.11	12.30 ± 5.25	9.30 ± 5.98
CK (U/L)	90.90 ± 15.91	110.00 ± 43.37	89.20 ± 27.59
ALB (g/L)	48.60 ± 1.78	48.30 ± 0.95	47.60 ± 1.35
TP (g/L)	74.20 ± 3.91	73.80 ± 2.25	72.60 ± 2.76
ALP(U/L)	65.00 ± 13.28	64.20 ± 22.00	58.20 ± 10.86
BUN (mmol/L)	3.25 ± 1.07	3.38 ± 1.11	3.29 ± 1.01
Creatinine (μmol/L)	56.30 ± 7.48	57.60 ± 13.29	61.40 ± 9.41
Glucose (mg/dL)	104.64 ± 6.11	104.75 ± 7.92	104.44 ± 6.07
TC (mg/dL)	174.63 ± 22.01	167.68 ± 19.50	170.77 ± 24.24
TG (mg/dL)	113.46 ± 64.73^a^	79.56 ± 19.04^ab^	71.69 ± 14.73^b^
HDL (mg/dL)	80.04 ± 22.38	87.95 ± 14.48	85.48 ± 18.62
LDL (mg/dL)	94.86 ± 21.64^a^	78.92 ± 11.70^b^	80.54 ± 11.36b^c^

Data are presented as mean ± standard deviation, with n = 10 subjects/group. Subjects were assessed 4 weeks after ECC training or ECC training combined with SA supplementation. One-way ANOVA was performed for statistical analysis, with *p* values <0.05 indicating statistical significance. Data in the same row (within group) followed by different letters (a, b) differed significantly at *p* < 0.05. AST, aspartate aminotransferase; ALT, alanine aminotransferase; CK, creatine kinase; ALB, albumin; TP, total protein; ALP, alkaline phosphatase; BUN, blood urea nitrogen; TC, total cholesterol; TG, triglycerides; HDL, high-density lipoprotein; LDL, low-density lipoprotein.

## 4 Discussion

The current study investigated the effects of SA supplementation on exercise performance among previously untrained female college students undergoing ECC training. Notably, our findings showed that during the aerobic capacity test, the ECC + SA group exhibited significantly longer aerobic exercise duration and time to exhaustion than did the ECC-only group, whereas no significant difference in maximal oxygen uptake (VO_2_ max) was observed between the two groups. Consistent with these findings, murine studies have shown that oral gavage of *R. rosea* extract prolongs swimming endurance in both 90-min unloaded and 5% body weight swim-to-exhaustion protocols without altering VO_2_ max, indicating that the observed endurance improvements are independent of changes in maximal oxygen uptake (Lee et al., 2009; Bang et al., 2019). Previous research has indicated that 14 weeks of altitude training combined with *Rhodiola*–*Cordyceps* supplementation significantly prolonged posttest exercise time in distance runners but left VO_2_max unchanged  (Chen et al., 2014). Similarly, other studies have revealed that 4 weeks of SA supplementation during eccentric-biased training extended the aerobic exercise time and produced antifatigue effects without altering maximal oxygen uptake (Lee et al., 2009; Xu and Li, 2012). Studies administering *R. rosea* extract for 7–30 days have also reported superior postexercise heart rate recovery compared to placebo, reflecting reductions in both physical and mental fatigue (Spasov et al., 2000; Xu and Li, 2012; Ivanova Stojcheva and Quintela, 2022; Tinsley et al., 2024). However, the present experiment found that the ECC + SA group showed no improvement in heart rate, likely due to differences in participant characteristics, dosing regimens, environmental conditions, and testing protocols relative to the aforementioned studies.

Our measurements of lower-extremity RSI during the repetitive DJ series showed that after 160 jumps, both the ECC and ECC + SA groups exhibited uniformly higher jump height and RSI across all 17 analyzed jumps (DJ_1_–DJ_160_) than did the SC group. These improvements parallel earlier findings obtained after 6 weeks of flywheel overload training (Vogt and Hoppeler, 2014) but were achieved here within just 4 weeks. Eccentric loading has been known to enlarge and preferentially recruit type II fibers, optimize muscle length–tension characteristics, and enhance muscle–tendon mechanical function (Douglas et al., 2017; Suchomel et al., 2019). Such adaptations likely support the superior jump height and, consequently, the greater RSI observed in both training groups considering that a higher jump height coupled with a shorter ground-contact time directly elevates RSI (Wang et al., 2021).

Notably, the ECC + SA group outperformed the ECC group in terms of RSI from DJ_120_ to DJ_160_. SA, a bioactive *Rhodiola* constituent with well-documented antifatigue properties, has been found to enhance glycogen storage, increase hemoglobin, reduce lactate accumulation, and augment antioxidant capacity (Lehnert et al., 2020), which may have preserved stretch–shortening cycle (SSC) efficiency late in the fatiguing bout. Given that RSI, together with leg-spring stiffness, reflects SSC conversion efficiency (Flanagan and Harrison, 2007), the SA-supported group may have maintained optimal leg stiffness and thus shortened contact time despite accumulating fatigue, allowing for a faster eccentric-to-concentric transition. Although jump height itself did not differ between the ECC and ECC + SA groups, the superior RSI implies a more effective mechanical utilization of stored elastic energy, independent of other factors, such as whole-body coordination. In summary, 4 weeks of flywheel eccentric training improved explosive SSC performance, whereas concurrent SA supplementation provided an additional late-stage RSI advantage, indicating a potent antifatigue role.

Several previous studies have confirmed that severe inflammatory reactions occur after acute eccentric exercise, which increases CK and other inflammatory factors in blood, thereby greatly accelerating the formation of micro-injuries in the skeletal muscle. CK, LDH, and aspartate aminotransferase in the blood have been used as biomarkers for muscle injuries (Clarkson and Hubal, 2002; González-Hernández et al., 2022; Gottschalk et al., 2025). Previous studies have demonstrated that continuous supplementation with *R. rosea* extract, or its principal active constituent, SA, for as long as 4 weeks, and even as briefly as 15 days, significantly attenuates the postexercise peak in serum CK, thereby protecting skeletal muscle from micro-damage (Abidov et al., 2004). After 4 weeks of SA supplementation, participants in the current study underwent an eccentric exercise-induced exhaustion test. Notably, we found that serum CK levels measured immediately after exhaustion were significantly lower in the ECC + SA group than in the SC group (*p* < 0.05). Moreover, posttest CK levels in the ECC + SA group were lower than baseline levels during the pretest, indicating that SA attenuated exercise-induced muscle damage. Taken together, these findings support the hypothesis that chronic SA supplementation may reduce the susceptibility to muscle injury following exhaustive exercise. Glucose and lactate are central substrates in whole-body energy metabolism, with plasma glucose serving as a primary fuel for ATP resynthesis during exercise. Therefore, maintaining euglycemia is essential for optimal physical performance. In the present study, blood glucose concentrations did not significantly differ between the pre- and post-exhaustion measurements and remained within the normal physiological range. These findings raise the possibility that SA supplementation facilitates the maintenance of euglycemia during exercise. By contrast, lactate is the terminal product of anaerobic glycolysis. During prolonged, high-intensity effort, glycolytic flux can outpace mitochondrial oxidative capacity. The resulting relative oxygen deficit drives the conversion of pyruvate to lactate, which accumulates within the working muscle and is eventually released into the circulation.

Consequently, rising lactate concentrations and the accompanying decline in blood pH contribute to neuromuscular fatigue and discomfort. Hence, blood lactate concentrations have been widely employed as a biochemical index of exercise load and as an indicator of the relative aerobic–anaerobic metabolic contribution. In the present study, lactate levels measured immediately after the exhaustion test were significantly higher than those measured at baseline in both groups (Figure 3A). However, no significant difference in lactate levels was observed between the two groups. Repeated bouts of eccentric contractions promote progressive lactate accumulation, hastening the onset of fatigue. In the present study, the eccentric flywheel squat test produced a sharp rise in blood lactate concentrations in both groups, with postexercise values being markedly higher than pre-exercise values. Importantly, the increase in lactate levels was attenuated in the ECC + SA group compared to the SC group, implying that chronic SA supplementation may modulate lactate kinetics during intense eccentric work, thereby mitigating exercise-induced fatigue.

Serum free fatty acid (FFA) concentrations provide a useful index of muscle fatigue considering that they mirror the size of intramuscular triglyceride stores and the rate at which these lipids are mobilized, thereby gauging the muscle’s capacity to access lipid fuel during exercise (Fritzen et al., 2020). Notably, our findings showed that plasma FFA concentrations were significantly higher in the ECC + SA group than in the SC group after exhaustion, suggesting that SA enhances FFA mobilization after exhaustive exercise and may expedite metabolic recovery.

Above all, the ECC + SA group preserved a higher late-stage RSI than the ECC group while exhibiting lower post-exercise lactate and ammonia and higher free-fatty-acid (FFA) levels. This pattern suggests that salidroside (SA) mitigated metabolic fatigue by enhancing lipid oxidation and reducing glycolytic stress. Pre-clinical work shows that SA activates AMPK and PPAR-α signalling, thereby up-regulating mitochondrial fatty-acid oxidation and delaying lactate accumulation (Panossian and Wikman, 2010). SA may also counter neuromuscular fatigue. Both training groups performed identical eccentric sessions for 4 weeks, inducing a repeated-bout effect that attenuates CK and FFA release (Howatson and Van Someren, 2008). Salidroside’s additional benefit may therefore be partially masked, yet the direction of change remained favourable.

It crosses the blood brain barrier, shifts the dopamine to serotonin ratio, and induces Hsp70 and other antioxidant defences, actions associated with preserved central drive under stress (Panossian et al., 2014). Peripherally, SA limits ROS-induced Ca^2+^ dys-regulation and Z-line disruption in eccentric-injury models (Huang et al., 2019; Kiriaev et al., 2023). Because we did not measure neural function or oxidative-stress markers, these mechanisms remain speculative. Future studies should combine surface EMG, transcranial magnetic stimulation, and oxidative-stress biomarkers to delineate SA’s central and peripheral effects more precisely.

### 4.1 Limitations

This experiment was conducted exclusively in young, recreationally active women; hence, extrapolation to males or highly trained athletes awaits further verification. Although an *a priori* calculation indicated that 24 participants per group were required to achieve 80% power, logistical constraints limited enrolment to 10 per group, thereby reducing statistical power and external validity. To isolate the interaction between a fixed eccentric load and salidroside, fly-wheel inertia was intentionally kept constant; the absence of progressive overload, however, may have attenuated longer-term neuromuscular adaptations. Finally, because no oxidative-stress or inflammatory biomarkers were assessed, the mechanistic interpretation of salidroside’s antifatigue and muscle-protective effects remains speculative and should be confirmed in future work that incorporates biochemical and neuromuscular assays.

## 5 Conclusion

The current study showed that 4 weeks of flywheel-based eccentric squat training enhanced neuromuscular and metabolic performance in previously untrained female students and that coadministration of SA produced additional benefits, particularly by mitigating fatigue. Both exercise groups showed improved time to exhaustion on a graded cycling test; however, the SA group (ECC + SA) displayed a further 8% gain over eccentric training alone without altering VO_2_ max or resting anthropometry. Repetitive DJ testing showed that eccentric training increased jump height and the RSI across 160 contacts, with SA supplementation conferring an extra 20%–33% improvement in the RSI during the most fatiguing final 40 jumps, indicating superior stretch–shortening cycle efficiency. Biochemical analysis showed that although eccentric exercise elevated lactate and ammonia in all groups. Postexercise ammonia did not differ significantly between groups, whereas CK was lower in ECC + SA compared with the SC group. Free fatty acids were higher in ECC + SA versus SC group, consistent with greater lipid mobilization.

Postexercise CK increased in the SC group but decreased by 26% with SA supplementation, suggesting reduced muscle damage. SA also augmented the availability of FFAs after exhaustive exercise, which was consistent with improved lipid mobilization and potential acceleration of metabolic recovery. Benefits in the lipid profile (reduced TG and LDL-C) were evident in both training groups, with greater effects having bene observed when combined with SA. Both training groups performed identical eccentric sessions for 4 weeks, inducing a repeated-bout effect that attenuates CK and FFA release, which may have partially masked salidroside’s additional benefits, though the direction of change remained favorable. Collectively, these findings demonstrate that short-term eccentric training alone enhanced explosive power and endurance capacity, whereas adjunct SA supplementation further limited muscle damage, moderated metabolite accumulation, and sustained SSC performance under high plyometric loads. Considering that our cohort comprised healthy young women from a sports university setting, future studies should verify whether similar ergogenic and protective effects extend to male athletes and other populations with varying training backgrounds.

## Data Availability

The original contributions presented in the study are included in the article/supplementary material, further inquiries can be directed to the corresponding author.

## Abbreviations

ANOVA, Analysis of variance; BMI, Body mass index; CK, Creatine kinase; DJ, Drop vertical jump; DVJ, Drop vertical jumps; FFA, Free fatty acid; FFM, Fat-free mass; HDL, High-density lipoprotein; IC, Initial contact; LDL, Low-density lipoprotein; RSI, Reactive strength index; SC, Sedentary placebo control; TC, Total cholesterol.
